# Monkeypox Outbreaks in 2022: Battling Another “Pandemic” of Misinformation

**DOI:** 10.3389/ijph.2022.1605149

**Published:** 2022-07-14

**Authors:** Farah Ennab, Faisal A. Nawaz, Kapil Narain, Goodluck Nchasi, Mohammad Yasir Essar, Michael G. Head, Rajeev K. Singla, Atanas G. Atanasov, Bairong Shen

**Affiliations:** ^1^ College of Medicine, Mohammed Bin Rashid University of Medicine and Health Sciences, Dubai, United Arab Emirates; ^2^ Nelson R. Mandela School of Medicine, University of KwaZulu-Natal, Durban, South Africa; ^3^ Department of Medicine, Catholic University of Health and Allied Sciences, Mwanza, Tanzania; ^4^ Kabul University of Medical Sciences, Kabul, Afghanistan; ^5^ Clinical Informatics Research Unit, Faculty of Medicine, University of Southampton, Southampton, United Kingdom; ^6^ Institutes for Systems Genetics, Frontiers Science Center for Disease-Related Molecular Network, West China Hospital, Sichuan University, Chengdu, China; ^7^ iGlobal Research and Publishing Foundation, New Delhi, India; ^8^ Ludwig Boltzmann Institute for Digital Health and Patient Safety, Medical University of Vienna, Vienna, Austria; ^9^ Institute of Genetics and Animal Biotechnology of the Polish Academy of Sciences, Jastrzebiec, Poland

**Keywords:** Monkeypox, pandemic, infodemic, outbreak, infection, infection control

Monkeypox is a virus that was formerly recognized as a rare zoonotic disease but has been noted as an emerging disease of concern where there are significant gaps in knowledge. In 2022, Monkeypox outbreaks are being concurrently reported in numerous non-endemic geographical areas, thus instigating a global wave of public health concern amid calls for urgent action from international authorities. As of the 8th of June 2022, about 1,285 laboratory-confirmed cases were detected in 28 regions across Africa, the Americas, and the European Region [1]. This virus is more commonly reported in West and Central Africa, where previous self-limiting outbreaks have had a mortality rate ranging between 1% and 15% [2]. However, the alarming surge of cases in 2022 with obvious multi-country community transmission is raising the alarm bells for monkeypox as a potential future global threat with unprecedented ramifications [3].

Misinformation is plentiful as Monkeypox virus cases are on the rise. The sensational nature of misinformation results in it being easily distributed and false beliefs perpetuated, further compounding the situation and causing widespread “misinfodemic” [4]. Misinformation is commonly-observed with high-profile disease outbreaks. The high degree of misinformation witnessed during the COVID-19 pandemic prompted the World Health Organization (WHO) to host the 1st WHO Infodemiology Conference in 2020 with international experts from various social and political backgrounds in order to manage this public health threat and establish a community of practice and research [5].

The origin of the Monkeypox virus, both in terms of the “first ever” monkeypox cases and also the origins of this 2022 outbreak, is widely questioned on social media. Both plausible and unlikely scenarios are discussed by a range of public commentators. One false claim is that the virus was manufactured and released intentionally from a laboratory in China. Furthermore, there are clearly unsubstantiated myths linking the Monkeypox virus to COVID-19 vaccination, stemming from the idea that the AstraZeneca vaccine utilized a chimpanzee viral vector. Monkeypox is a pox virus whilst the AstraZeneca vaccine is based on a weakened and altered adenovirus vector that cannot infect humans [6]. In addition, various social media platforms have been riddled with false narratives; one example is there from many Facebook posts, claiming that the current rash images that are broadcasted in mainstream media are merely a production of edited and reused photos from previous outbreaks in African countries, suggesting that the current outbreaks in 2022 are a hoax [7]. Another claim that was widely shared on Twitter was the suggestion that the Monkeypox virus was intentionally rolled out by powerful entities in the private health sector in order to benefit the pharmaceutical companies that manufacture vaccines and medical therapies [2].

Misinformation can drive stigma [8]. Given that many of the cases reported so far are linked to sexual networks, the public health messaging has to strike a balance between highlighting the transmission within highest-risk populations, whilst also encouraging potential new cases to report to a health facility. Strategies that can mitigate any impact of the rising Monkeypox misinfodemic are warranted. This should involve multi-faceted initiatives across diverse global health stakeholders. Community engagement across social media is vital to combat this challenge. Online awareness campaigns on sharing reputable resources related to Monkeypox transmission, treatment, and vaccination can help create an accessible source of reliable information for the public. This effort also involves collaborations between public health entities and social media platforms in actively promoting reliable information on the disease outbreak. Public health leaders, as well as social influencers, can play a strategic role in advocating for Monkeypox awareness on social media. A similar approach is recommended to target mega-event platforms such as the FIFA World Cup 2022 as “incubators” for raising awareness about the importance of COVID-19 vaccinations [9].

However, encouraging the spread of accurate information on social media is not enough to completely counter any harm of misinformation. An ongoing review of social media is necessary to identify evolving trends, new rumors, and key sources of misinformation. Introducing a flagging system for self-reporting suspicious posts related to Monkeypox misinformation can help foster responsible behaviors within the online community. Additionally, research is warranted for understanding the impact of misinformation on disease outbreaks *via* social media analysis of user behavioral trends. This can aid in exploring major causes of misinformation as well as predict disease outbreaks as seen during the COVID-19 pandemic [10].

Outbreaks of emerging infections like Monkeypox will continue to occur, and the general public will continue to seek information from easily accessible internet resources. Online misinformation leads to unwarranted public concern and may exacerbate the outbreak of emerging infectious diseases due to improper public health guidance. Multi-faceted efforts are warranted between governments, social media platforms, and community leaders in combatting this public health crisis.
